# Behavioral Phenotyping of Bbs6 and Bbs8 Knockout Mice Reveals Major Alterations in Communication and Anxiety

**DOI:** 10.3390/ijms232314506

**Published:** 2022-11-22

**Authors:** Nathalie Rödig, Kristin Sellmann, Malena dos Santos Guilherme, Vu Thu Thuy Nguyen, Dirk Cleppien, Albrecht Stroh, Helen Louise May-Simera, Kristina Endres

**Affiliations:** 1 Department of Psychiatry and Psychotherapy, University Medical Center of the Johannes Gutenberg-University Mainz, 55131 Mainz, Germany; 2 Leibniz Institute for Resilience Research, 55122 Mainz, Germany; 3 Institute of Pathophysiology, University Medical Center of the Johannes Gutenberg-University Mainz, 55128 Mainz, Germany; 4 Cilia Cell Biology, Institute of Molecular Physiology, Johannes Gutenberg-University, 55128 Mainz, Germany

**Keywords:** ADAM10, Bardet-Biedl syndrome, behavior, hypothalamus, Lsamp, MRI, primary cilium

## Abstract

The primary cilium is an organelle with a central role in cellular signal perception. Mutations in genes that encode cilia-associated proteins result in a collection of human syndromes collectively termed ciliopathies. Of these, the Bardet-Biedl syndrome (BBS) is considered one of the archetypical ciliopathies, as patients exhibit virtually all respective clinical phenotypes, such as pathological changes of the retina or the kidney. However, the behavioral phenotype associated with ciliary dysfunction has received little attention thus far. Here, we extensively characterized the behavior of two rodent models of BBS, Bbs6/Mkks, and Bbs8/Ttc8 knockout mice concerning social behavior, anxiety, and cognitive abilities. While learning tasks remained unaffected due to the genotype, we observed diminished social behavior and altered communication. Additionally, Bbs knockout mice displayed reduced anxiety. This was not due to altered adrenal gland function or corticosterone serum levels. However, hypothalamic expression of Lsamp, the limbic system associated protein, and Adam10, a protease acting on Lsamp, were reduced. This was accompanied by changes in characteristics of adult hypothalamic neurosphere cultures. In conclusion, we provide evidence that behavioral changes in Bbs knockout mice are mainly found in social and anxiety traits and might be based on an altered architecture of the hypothalamus.

## 1. Introduction

Primary cilia are microtubule-based appendages extending from the surface of most eukaryotic cells. In contrast to motile cilia, which serve to move fluid over membrane surfaces and defects of which have long been recognized as a cause of numerous medical conditions [1], the consequences of primary cilia dysfunction were only identified at the turn of the millennium. We now know that primary cilia function as specialized signaling organelles, disruption of which causes a range of diseases that come under the umbrella term ciliopathies [2]. Ciliopathies are characterized by a multitude of overlapping clinical phenotypes involving numerous organs and tissues, which can present individually, as in the case of non-syndromic ciliopathies, or in combination, as in the case of syndromic ciliopathies. The most common phenotypes include retinal degeneration, kidney disease, and obesity. It has been estimated that 1 in 1000 individuals worldwide is thought to be affected by a ciliopathy [3].

The Bardet-Biedl syndrome (BBS) is considered the archetypical ciliopathy, since BBS patients exhibit all phenotypes associated with primary cilia dysfunction. It was first described by two physicians over 100 years ago [4,5], and is characterized by retinitis pigmentosa, postaxial polydactyly, obesity, and urogenital and renal malformations, among other features [6]. The disease is caused by genetic mutations in the so-called BBS genes, all of which affect cilia function. In total, 23 BBS genes have been identified so far, many of which function together in multi-protein complexes [7]. The BBSome (BBS1, BBS2, BBS4, BBS5, BBS7, BBS8, BBS9, and BBS18) plays a critical role in regulating cilia protein trafficking, while the chaperonin-like complex (BBS6, BBS10, and BBS12) is required for the correct folding of the BBSome. Since BBS is the flagship ciliopathy, it is a good model to study underlying disease mechanisms affecting all primary cilia-associated phenotypes. There have been several different BBS mouse models generated, which recapitulate the patient phenotypes [8,9,10,11,12,13,14].

While retinal degeneration and kidney abnormalities have been examined extensively, cognitive and behavioral deficits have mostly been neglected, even though patients exhibit behavioral phenotypes, including secluded behavior, anxious or depressive traits, and difficulties in socialization [6,7,15]. However, these observations might also be attributed to society’s reaction towards manifestations of the syndrome, such as obesity, awkwardness, or language deficits [6,15].

Only a few clinical studies have focused on the social–behavior defects in BBS patients [7,8]. Moreover, limited data are available on respective mouse models for BBS concerning behavior, even though these models present high congruency to the human syndrome regarding other symptoms, such as visual impairment (e.g., [9,12]). The few studies available so far report on anxiety, reduced social dominance, and associative learning defects in Bbs2 and 4 knockout mice, in part due to impaired neurogenesis [8,9,13]. More recently, a mouse model for Ccdc28b, a modifier of BBS, was shown to display obsessive compulsive and mild social behavioral phenotypes [14].

Therefore, in this study, we analyze in-depth social behavior, as well as anxiety and cognitive functionality, in two ciliopathy mouse models, namely, the Bbs6/Mkks and Bbs8/Ttc8 knockout mice [10,11]. BBS8 is an integral part of the BBSome protein complex that controls the transport of proteins within the primary cilium [16], and BBS6 is a component of the chaperonin-like complex, which assists in the folding of the BBSome. Deficiency of either protein in mice does not result in total loss of the primary cilium, but in a complex pattern of altered ciliary appearance. Abnormal ciliation in terms of the percentage of ciliated cells, as well as ciliary axonemal length, have been observed in a variety of different tissues, such as the olfactory epithelium and retinal pigmental epithelium [11] [17,18]. Since ciliation is intricately connected to cell and tissue homeostasis and function, defects can subsequently be assumed to have a wide spectrum of effects. We analyzed both sexes of Bbs6 and 8 knockout mice for their behavior and, in this regard, we investigated animals at the comparable early age between 4–8 weeks to avoid the influence of progressive sensory deficits on behavioral tests. These studies shed light on the complex behavioral traits caused by ciliary dysfunction and began to decipher the underlying molecular mechanisms, which could lead to a more comprehensive understanding of the patient phenotype.

## 2. Results

### 2.1. Enlarged Lateral Ventricles in Bbs6 and 8 Knockout Mice

**Figure 1:**
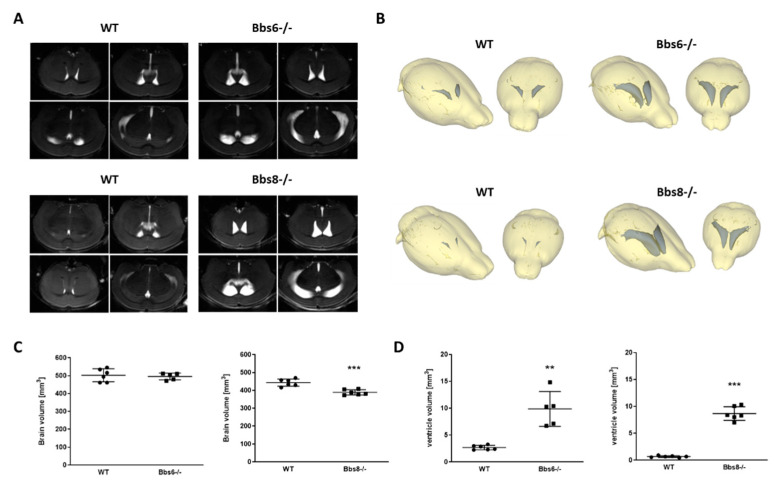
Increased volume of lateral ventricles in Bbs6 and Bbs8 knockout mice. (**A**) Mice were anesthetized with isoflurane and subjected to MRI (*n* = 6 for wild type (WT) and 5 for Bbs6−/−; *n* = 6 for WT and Bbs8−/−; 40% females in the Bbs6−/− group, for all other groups 50% females). (**B**) MRI images were used for a three-dimensional modelling of the brain structure and volumetric analysis of brain and lateral ventricle size. Quantitation of brain (**C**) and ventricle volumes (sum of both lateral ventricles (**D**)). Student’s unpaired *t*-test was used for statistical analysis (** *p* < 0.01; *** *p* < 0.0001).

Aberrant brain anatomy has been described for Bbs3 knockout mice with severe hydrocephalus, reduced cortex thickness, and size of hippocampus and corpus callosum [19]. Therefore, we firstly assessed impact of the ablation of either gene by MRI. Whole brain volume was unaffected in Bbs6 knockout mice, while Bbs8 knockout animals revealed a mean loss of 12% (Figure 1A–C). For both BBS models, enlarged lateral ventricles were observed (Figure 1A,B,D; >350% for Bbs6−/−; >1200% for Bbs8−/− in comparison to the respective wild type littermates). Such brain anatomical structural changes may already indicate altered functionality, as has been observed in other genetic mouse lines [20,21,22].

### 2.2. Behavioral Testing Reveals Early Olfactory Deficits in Bbs8 Knockout Mice

**Figure 2:**
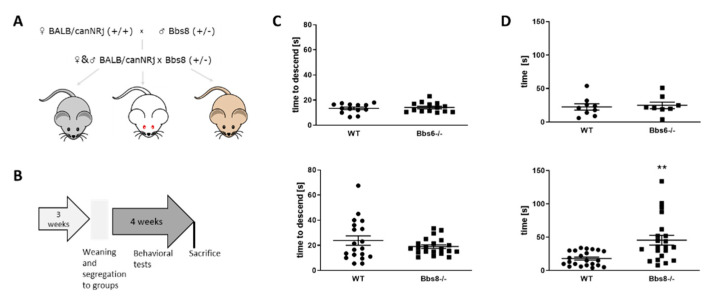
Behavioral testing scheme and test-prerequisites. (**A**) Breeding scheme of Bbs8 knockout mice. Male Bbs8−/+ mice were crossed to BALB/canNRj female mice and the derived F1 offspring were mated to obtain F2 (Bbs8−/− or Bbs8+/+). Only F2 animals were used for the investigations. (**B**) Timeline of behavioral testing. Sacrifice was conducted at the age of 8 weeks. (**C**) Locomotor activity assessed by the pole test (*n* = 13 for WT and *n* = 14 for Bbs6−/−; *n* = 19 for WT and *n* = 21 for Bbs8−/−). (**D**) Olfactory testing of knockout mice (*n* = 8 for WT and *n* = 9 for Bbs6−/−; *n* = 22 for WT and *n* = 21 for Bbs8−/−). Data for the third test day (fully buried palatable food) are shown. Student’s unpaired *t*-test was used for statistical analysis (** *p* < 0.01).

Bbs6 knockout mice were maintained on a homogenous J129 background. However, Bbs8 knockout mice had to be crossed to BALB/canNRj mice to obtain a mixed genetic background, which allows the knockout to survive better. This resulted in three different phenotypes with different fur and eye color, as depicted in Figure 2A. To exclude that these differences might have an impact on the outcome of our behavioral tests, we made sure that the composition of each group tested —wild type and Bbs8 knockout—equally contained tyr mutated red-eyed animals [23] and, also, fur color types (for detailed group composition see Supplementary Table S1).

Mice were subjected to a battery of behavioral tests within four weeks (see Supplementary Table S2), including assessment of locomotor function in the pole test and olfactory function by measuring the time to find a hidden palatable piece of food (Figure 2B). No general locomotor deficits could be observed (Figure 2C), while olfactory sensitivity seemed decreased in the Bbs8 knockout mice already at around two months of age (Figure 2D), which is in line with a previous study, which found that loss of Bbs8 on another genetic back-ground perturbs olfactory function, protein localization, and axon targeting [11]. Both, locomotion as well as olfaction, are important clues for optimal performance in the subsequently conducted behavioral tasks. Therefore, the deprivation of the olfactory sense has to be considered when evaluating the following results for Bbs8 knockout mice.

### 2.3. Socialization Is Unaffected in BBS Deficient Mice but Dominance-Related Behavior Is Reduced

**Figure 3:**
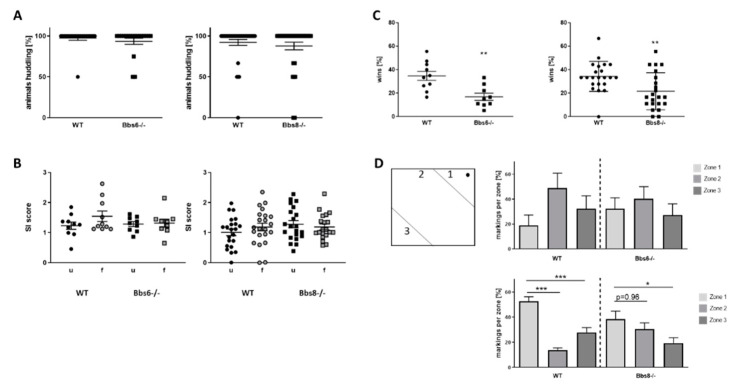
Social behavior of Bbs6 and Bbs8 knockout mice. (**A**) The number of mice huddling while sleeping was assessed (*n* = 19 observed events for WT and Bbs6−/− and *n* = 36 and 34 for WT and Bbs8−/−). (**B**) Social interaction with an unfamiliar (u) or a familiar (f) conspecific within a plexiglas cylinder was measured and set in relation to the time used to inspect an empty cylinder (social interaction (SI) score) (*n* = 10 for WT and *n* = 9 for Bbs6−/−; *n* = 22 for WT and Bbs8−/−). (**C**) Dominance behavior was tested in the tube test with cage-mates (*n* = 10 for WT and *n* = 9 for Bbs6−/−; *n* = 22 for WT and Bbs8−/−). (**D**) Urine scent marking was measured in the open field arena by stimulating male mice with pro-estrous female urine (wild type mouse). (*n* = 16 events for WT and *n* = 21 events for Bbs6−/−; *n* = 36 events for WT and *n* = 25 events for Bbs8−/−). The scheme indicates the zone distribution within the open field arena and the applied female urine (black dot). Student’s unpaired *t*-test was used for statistical analysis (** *p* < 0.01) or one-way ANOVA with Sidak’s multiple comparison test (* *p* < 0.05; ** *p* < 0.01; *** *p* < 0.001)).

Next, social behavior within a known group of conspecifics was tested, and the number of each genotype found huddling while sleeping in a group was assessed (huddling, Figure 3A). Here, no difference was observed between wild type and knockout animals. Additionally, inspection of conspecifics was not affected by either knockouts: interaction-time, spent with either an unfamiliar or a familiar same-sex mouse, was indifferent to the values measured for wild type animals (Figure 3B). In contrast, a strong phenotype occurred when investigating dominance: while wild type mice of both sex won about 40% of all confrontations in the tube test, this was decreased to about 20% in the knockout animals (Figure 3C). Interest in mating, which is eventually regulated by dominance, can be monitored via the urine scent marking test in males [24]. The interest is indicated by the mouse via marking a region with urine in the vicinity of an applied droplet of pro-estrous female urine (zone 1, see schematic in Figure 3D). For the Bbs8 wild type, markings were significantly favored to occur in zone 1, as expected, while in the Bbs8 knockout males, this zone was less favored and was not significantly preferred to zone 2. Interestingly, the data from the Bbs6 line displayed no definite conclusion since the respective wild type did not perform as expected (preference of zone 1). However, the marking pattern seems more random in the knockouts as compared to the wild type mice, who had a slight preference for zone 2.

### 2.4. Diminished Communicational Skills in BBS Knockout Mice

**Figure 4:**
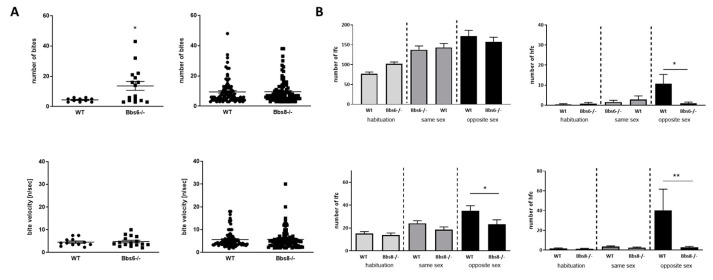
Communication characteristics in Bbs6 and Bbs8 knockout mice. (**A**) Orofacial characteristics as assessed by bite/event counting and bite velocity during the pasta gnawing test (*n* = 10 for WT and 16 for Bbs6−/−; *n* = 88 per Wt or Bbs8−/−). (**B**) Ultrasonic vocalization evoked by neutral, unused (habituation phase), same sex or opposite sex bedding material (lfc: low frequency calls; hfc: high frequency calls) (*n* = 9–10 animals per WT or Bbs6−/−; *n* = 22 animals per WT or Bbs8−/−). Student’s unpaired *t*-test was used for statistical analysis (* *p* < 0.05; ** *p* < 0.01).

Social behavior in mice is accompanied by and based on ultrasonic vocalization [25]. Therefore, we analyzed the orofacial anatomical prerequisite of utterance by the pasta gnawing test (Figure 4A). Both knockouts showed a comparable ability to process the piece of pasta provided, as their wild type littermates. Bite number per event was elevated in Bbs6 knockout animals. Therefore, no anatomical hindrance seems to be present that might interfere with vocalization, even if anatomical structures involved in this are not well characterized for mice. Exposition to neutral, unused bedding material, as well as to same-sex used bedding material, neither affected low frequency nor high frequency calls in the knockouts compared to the respective wild type mice (Figure 4B). However, low frequency calls were significantly reduced in Bbs8 knockout mice by opposite sex-derived bedding material. This effect was also observed in tendency in the Bbs6 knockouts but did not reach statistical significance (*p* = 0.194). The effect was even more pronounced when numbers of high frequency calls were counted: both knockouts showed a strong reduction in the positively annotated vocalization type. This indicates that, while the anatomical prerequisite seems to be there as shown by the pasta gnawing test, the knockouts display reduced communicational interaction.

### 2.5. Cognitive Performance in Bbs6 and Bbs8 Knockout Mice Is Largely Unaffected

**Figure 5:**
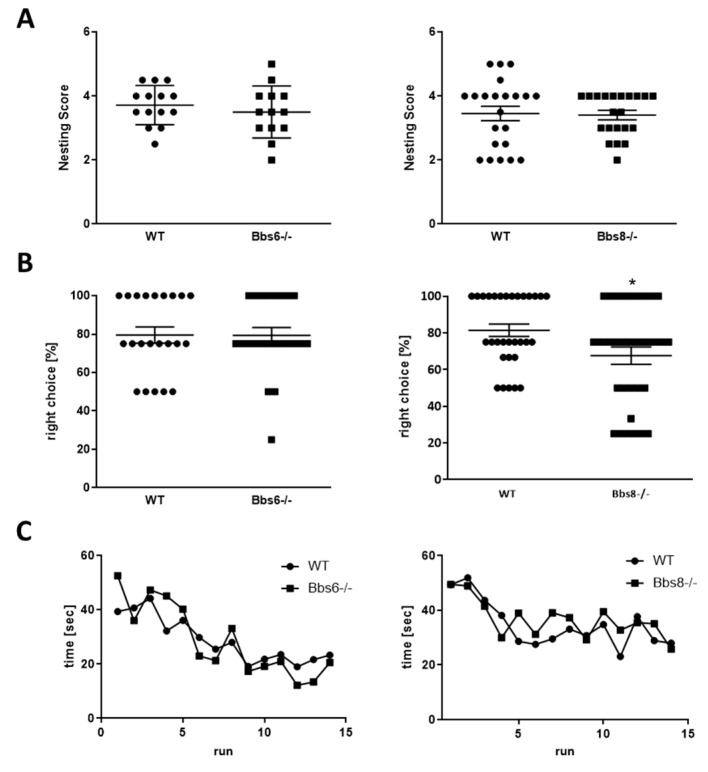
Cognitive performance in Bbs6 and Bbs8 knockout mice. (**A**) Quantitation of nest building ability (*n* = 14 for WT, *n* = 13 for Bbs6−/−; *n* = 22 for WT and Bbs8−/−). (**B**) The T-maze test was performed on two different days. Means of both test days are presented (*n* = 22 for WT and *n* = 23 test events for Bbs6−/−; *n* = 32 for WT and Bbs8−/−). (**C**) The radial arm water maze was conducted over two days with an overnight rest after the 8th trial (*n* = 10 WT and *n* = 9 Bbs6−/− animals; *n* = 22 WT and Bbs8−/− animals). Student’s unpaired *t*-test or multiple *t*-test were used for statistical analysis (* *p* < 0.05).

Cognitive performance is highly variable amongst BBS patients, ranging from severe mental handicap to average intelligence in about 30% of the patients to even high intellectual abilities in a small subgroup [26]. This led us—together with the observed brain anomalies—to evaluate spatial orientation and learning (Figure 5).

The nesting test, that evaluates hippocampal integrity, showed that both knockouts performed similarly as their wild type littermates (Figure 5A). Further, the non-integrated amount of material was comparable between the genotypes. In the T-maze test, Bbs6 knockout mice were indistinguishable from the wild type (Figure 5B), while Bbs8 knockout mice displayed a 14% reduction of correct choice performance. However, with 68% of choices correct, they still show a high functionality above the 50% coincidence ratio. Moreover, the learning curves of wild types and Bbs knockout mice in the radial arm water maze were comparable (Figure 5C). Thus, both knockouts showed no significant cognitively impaired behavior at this early age.

### 2.6. Bbs6 and Bbs8 Knockout Mice Display Reduced Anxiety in Non-Social Situations

**Figure 6:**
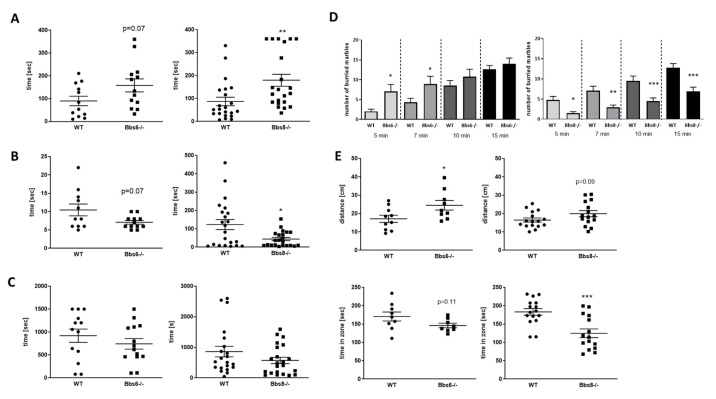
Anxiety-related behavior in Bbs6 and Bbs8 knockout mice. (**A**) Latency to immobile state in the forced swim test (*n* = 12 for WT and *n* = 13 for Bbs6−/−; *n* = 22 for WT and Bbs8−/−). (**B**) Time to escape the box in the neophobia test (*n* = 11 for WT and *n* = 10 for Bbs6−/−; *n* = 22 for WT and Bbs8−/−). (**C**) Novelty induced suppression of feeding was measured in a modified open field arena (*n* = 13 for WT and *n* = 14 for Bbs6−/−; *n* = 22 for WT and Bbs8−/−). (**D**) The number of buried marbles per time period is shown (*n* = 13 for WT and *n* = 14 for Bbs6−/−; *n* = 22 for WT and Bbs8−/−). (**E**) Distance travelled within the first 5 min and time spent in the corners (first 5 min) in the open field arena (*n* = 8 for WT and *n* = 9 for Bbs6−/−; *n* = 22 for WT and Bbs8−/−). Examples of heat maps for movement profile visualization are given in Supplementary Figure S1. Student’s unpaired *t*-test was used for statistical analysis (* *p* < 0.05; ** *p* < 0.01; *** *p* < 0.001).

Social status in mice has recently been correlated to anxiety during stressful experiences. For example, dominant females have been found to be less anxious when exposed to mild stress [27]. We therefore analyzed depression-like and anxiety-related behavior (Figure 6). Astonishingly, we found that both BBS knockouts were more resistant in the forced swim test and displayed prolonged time to immobility (Figure 6A), which was statistically significant for Bbs8 knockout mice. A similar observation could be made in the neophobia test, where Bbs8 knockout mice entered significantly faster into the aversive arena, and Bbs6 knockout mice at least showed a trend toward the same effect (Figure 6B).

In the novelty induced suppression of feeding test, both knockouts revealed a trend toward reduced grabbing and feeding of palatable food, which, however, did not reach statistical significance (Figure 6C, *p* = 0.34 and 0.16). The marble burying test was initially considered to be a measure of anxiety, based on the finding that this behavior can be reduced by administration of anxiolytic drugs (reviewed in [28]). However, it could not be demonstrated that it presents a defensive response to a novel stimulus (e.g., [29]) but, rather, a test to measure compulsive digging behavior [30]. Interestingly, while all other tests performed so far indicated similar results for Bbs6 and Bbs8 knockout mice with a milder manifestation in the Bbs6-deficient animals, here, we observed the reverse (Figure 6D). While Bbs6 knockout mice displayed a significantly increased burying behavior as compared to their wild type littermates, Bbs8 knockouts showed a significantly decreased activity. Behavior in the open field arena, which can also be used to analyze anxiety, revealed increased distance travelled during the observation period, along with a reduced sojourn in the corners in both genotypes as compared to the respective wild types (Figure 6E). In summary, this provides evidence for reduced anxiety of both Bbs-deficient mouse strains—at least in non-social contexts.

### 2.7. Impact of Bbs Protein Deficiency on the HPA Axis

**Figure 7:**
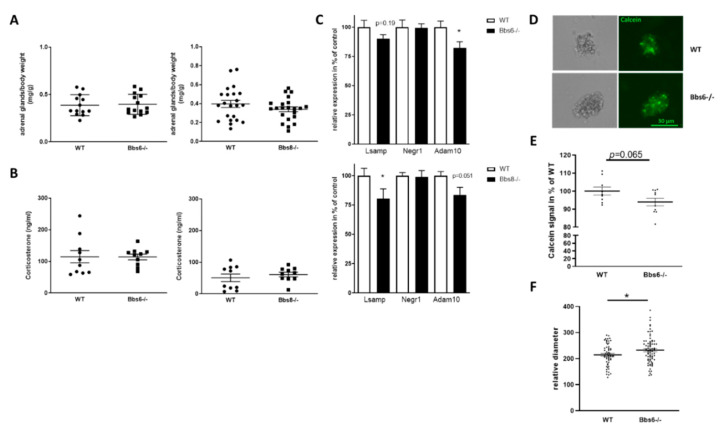
Effects on primary cilia defects on HPA axis. (**A**) Adrenal glands were weight pairwise during dissection and normalized to body weight (*n* = 13 for WT and *n* = 14 for Bbs6−/−; *n* = 22 for WT and Bbs8−/−). (**B**) Corticosterone was measured by HPLC in serum samples (*n* = 10 for all groups). Student’s unpaired *t*-test was used for statistical analysis. (**C**) Expression of IgLON members and Adam10 in the hypothalamus, analyzed by qRT-PCR (*n* = 5-6 for WT and for Bbs6−/−; *n* = 5–6 for WT and Bbs8−/−). (**D**) Hypothalamic neurospheres of 10–12-week-old animals were cultivated up to day seven in vitro (*n* = 3 for wild type; *n* = 4 for Bbs6−/−). Three wells per animal were stained by Calcein to demonstrate viability of the cell aggregates. Exemplary images are shown. (**E**) Fluorescence intensity was measured in lysates of the neurosphere cultures and normalized to the mean value obtained from wild type animals. (**F**) The horizontal diameter of randomly chosen neurospheres was assessed by ImageJ. Student’s unpaired *t*-test was used for statistical analysis (*n* = 5–6 animals per group; * *p* < 0.05).

In summary, the results of behavioral tests suggested that Bbs6 and Bbs8 knockout mice have a deficient opposite-sex sociability along with exhibiting reduced dominance and decreased anxiety. Such phenotypes might be based on alterations in the HPA axis functionality. Therefore, we analyzed adrenal gland weights at the end of the experiments and assessed corticosterone serum levels (Figure 7A,B).

Neither adrenal gland weights nor corticosterone serum levels deviated between the respective wild type and the Bbs knockout animals, hinting at an unaltered adrenal function. However, this only reflects the peripheral part of the HPA axis. To analyze whether the CNS-associated HPA axis is affected by either genotype, we assessed mRNA levels of two members of the immunoglobulin superfamily cell adhesion molecule subgroup IgLON in the hypothalamus. The neuronal growth regulator 1 (encoded by the gene Negr1) and the limbic system-associated membrane protein (encoded by Lsamp) have been shown to be expressed in the hypothalamus and to be involved in anxiety and depression-like behavior regulation [31,32]. While Negr1 mRNA was unaffected in both Bbs knockout lines, as compared to their wild type littermates, Lsamp mRNA level was decreased, reaching statistical significance in Bbs8 knockout mice (Figure 7C). Both, Negr1 and Lsamp, have been identified as Adam10 (A disintegrin and metalloproteinase 10) substrates [33,34]. Therefore, we also measured mRNA level of this metalloproteinase: in both Bbs protein deficient mouse lines, the levels were reduced (Figure 7C), hinting at an altered ectodomain shedding of substrates present in the hypothalamus. The IgLONs are important for dendritic arborization but have additionally been found to interfere with neurogenesis [35,36]. Moreover, Adam10 was demonstrated to affect embryonic neurosphere organization [37]. We therefore generated adult hypothalamic neurosphere cultures from wild type and Bbs6 knockout mice (Figure 7D) and assessed viability via calcein stain. This revealed a slight decrease in signal intensity in the knockout animals that did not reach statistical significance. Nevertheless, when comparing the diameter of neurospheres, an increased value was obtained for Bbs6−/− mice, pointing at an altered neurogenic capacity.

## 3. Discussion

Here we provide a side-by-side detailed behavioral analysis of mice with knockout of either a BBSome component (Bbs8) or an associated chaperonine-like component (Bbs6). In summary, both mouse strains revealed defects in social behavior and an anxiolytic phenotype with a more pronounced manifestation in the Bbs8−/− mice. These behavioral phenotypic traits were associated with neurological–anatomical abnormalities.

### 3.1. Bbs6 and 8 Knockout Mice Show Ventriculomegaly

Ventriculomegaly or hydrocephalus is elicited by an increased volume of cerebrospinal fluid (CSF) in the ventricular system of the brain. In children, this leads to cognitive disorders, including attention, executive, memory, visual, spatial or linguistic dysfunction (e.g., [38]). Motile cilia, which adorn the ependymal lining of brain ventricles, are master regulators of CSF flow (reviewed in [39,40]). The primary cilium has already been shown to modulate the development and function of ependymal motile cilia, which ultimately contributes to hydrocephalus, often found in ciliary mutant mice. Examples include Bbs1M390R/M390R mice [41], Bbs2−/−, Bbs4−/−, Bbs6−/− mice, and Bbs1M390R/M390R knockin mutant mice [42]. Hydrocephalus has also been reported in BBS patients [43,44]. This might be due to disturbed signaling in the choroid plexus epithelial cells, which express grouped cilia and seem to be important in chemosensing and, with this, in CSF production [45,46]. Here, we confirmed ventriculomegaly for Bbs6−/− mice and found the ventricle size to be elevated more than threefold. This is lower as compared to previous measurements (about 25-fold, [42]), but it might be due to the relatively young age of mice used in this study (up to eight weeks vs. three-and-a-half to six months of age). Additionally, we report a more severe ventriculomegaly in Bbs8−/− mice: ventricle size was increased by about 12-fold. In a previous report, it was said that “congenital Bbs8−/− mice develop hydrocephalus in the late prenatal/early postnatal period” [47]. Unfortunately, no quantitation was provided in this previous study. The strain background could also play a role in the pronunciation of the phenotype, since each of the mouse lines was bred on a different background (J129-for Bbs6 versus J129 backcross with BALB/canNRj for Bbs8). However, the stronger anatomical phenotype in the Bbs8−/− mice also correlated with a more pronounced behavioral phenotype. In general, different mouse strains can show different manifestations of behavioral traits (for example, [48]). Nevertheless, this observation might depend on different functions and expression levels of either BBS protein: the BBSome component Bbs8 revealed a comparable mRNA level in the brains of adult mice, as compared with the chaperonine-like component Bbs6 (0.17 vs. 0.2, normalized to Gapdh, [49]). However, in these investigations, whole brain RNA was analyzed, and local differences might be even more pronounced. Additionally, the loss of Bbs8 also reduced Bbs6 transcript levels in the adult murine brain, while loss of Bbs6 did not affect other BBSome transcripts such as Bbs8.

### 3.2. Behavioral Phenotypes of Bbs6 and 8 Knockout Mice

Locomotion was not affected in both mouse strains investigated and, to the best of our knowledge, no reports exist on altered muscular characteristics or locomotive performance in BBS patients. Thus, behavioral tests were not corroborated by locomotor differences between the knockout mice and their respective wild type controls. In contrast, hyposmia has been shown to be part of the clinical syndromes in human BBS patients [50,51,52] and has even been correlated with local atrophy of brain gray matter (e.g., left middle temporal gyrus, right hippocampus, and amygdala, [53]). While patients did not display general brain atrophy in a MRI study (BBS 1,497,830 mm^3^; controls 1,543,205 mm^3^; *p* = 0.2, [53]), we observed brain volume reduction of 12% in Bbs8−/− mice and also olfactory deficits, while Bbs6−/− mice neither had deficits in olfaction nor showed brain atrophy at eight weeks. For some Bbs mouse models, olfactory deficits have already been described (Bbs1 and 4−/−, [52]; Bbs2 and Bbs4−/−, [9]). Specifically, for the Bbs8−/−, a five-fold reduction in odorant-evoked activity was previously recorded by electroolfactogram measurements [11] for one to four months old animals. This could be confirmed by the behavioral olfaction test within our study, which indicated a 2.5-fold lower performance in the Bbs8−/− mice, as compared to wild type littermates. For Bbs6 mouse models, olfactory dysfunction has already been described [8,10]. However, the animals displaying the impaired sensory function were 16 to 34 weeks old—more than double the age of the mice investigated in this study. Thus, onset of olfactory deficits seems to occur later in Bbs6−/− mice, as compared to Bbs8−/− mice, and this underlines the more pronounced phenotype of the latter. It has to be kept in mind that olfaction is an important behavioral driver in rodents and, thus, observed phenotypes in the Bbs8−/− mice might also depend on the olfactory impairment.

To test for social behavior, huddling in-cage and social interaction with known and unknown conspecifics was tested. Neither of the two knockouts revealed any obvious deficits. Therefore, approach and contact to other individuals was not impaired. However, a reduced dominance behavior in the tube-test in both models and reduced urine scent-elicited marking behavior in male Bbs8−/− mice was observed. A homozygous knockout of Bbs2 has been reported to result in more submissive behavior, which was also tested by the tube test [9]: 86% of all tests were won by the wild type conspecifics. A comparable elevated submissive phenotype was described for Bbs4−/− mice [13]. Our own results displayed twice as high of a tendency to win by the wild type, as compared to each Bbs knockout. In our set-up, we used cage-mates for the testing, while this is not clear for the previous reports. Moreover, Bbs6 and 8−/− mice were aged eight weeks, whereas the exact age of the investigated mice in the report on Bbs2 and 4−/− mice is not clear (probably seven or 10 weeks, [9,13]). More recently, knockout mice for coiled–coil domain-containing protein 28B (Ccdc28b), a modifier of cilia length [54], were also reported to show social defects: while anogenital contacts in a reciprocal social interaction were not reduced, nose–nose and side sniffing was found less in the knockout, as compared to the wild type [55]. In a study on human BBS patients aged between two and 61 years, most participants (80%) were reported to show social deficits [56]. However, the study included self-reports and reports by parents (for those aged below 22), which might interfere with the outcome. An earlier report by Barnett and colleagues [15] comparably described that externalizing behavior, such as aggression, was seldomly observed in a cohort of 21 BBS children, but problems with social behavior were frequent. However, it is not clear for human patients if social disabilities are primarily caused by the ciliopathy or are secondary effects elicited by sensory deficits or social ostracization. They might also be based on difficulties with language, which is indicated in our study by the reduced vocalization of Bbs6 and 8−/− mice. Orofacial defects have been described for human BBS patients (reviewed in [57]). Thus, we checked functionality of the orofacial musculature of the mice by measuring biting events. These revealed no obvious deficits. Interestingly, the deficits in vocalization due to exposition to used bedding material only occurred in opposite sex constellations, but not in same-sex situations, and were more pronounced in high frequency calls. These present positive reactions to a conspecific and might reflect the internalizing behavior abnormalities found also in BBS patients. In a small study on children affected by BBS, basal serum testosterone concentration and testosterone response to human chorionic gonadotropin were lower as compared to controls [58], and hypogonadism, genitourinary abnormalities, and infertility are also amongst the symptoms of BBS (e.g., reviewed in [59]). Therefore, we cannot exclude that deficits in sexual performance have contributed to the outcome of vocalization recording, as we did not measure sexual hormones.

Reports on cognitive performance in human BBS patients are quite diverse: from 21 investigated children, for example, three showed unimpaired IQ, eleven displayed mental retardation, and 10 had mild mental retardation [15]. A study on adult patients (*n* = 34, aged 17–53 years, [60]) documented that 26% had mental retardation, but one patient even showed an IQ above 120. In another study, e.g., mean full scale IQ of patients, mean working memory index, and mean processing speed index fell more than 1 SD below the normal mean [56]. Another study reported that, while mean intellectual functioning of participants was 1.5 standard deviations below normal, the majority of participants did not indicate an intellectual disability [61]. In mice, Bbs1-deficient animals showed defects in long-term context-dependent fear conditioning but normal acquisition behavior [14], while Bbs4−/− mice showed reduced context but also cued fear memory [62]. Here, we tested learning and hippocampal function via the nesting test, the T-maze, and the radial arm water maze, and we were not able to find deficits in the young Bbs6 and Bbs8−/− mice. This might be one of the reasons why BBS patients display diverse intellectual abilities even if no correlation between IQ and the mutated BBS locus was observed so far [56]. However, this might also be due to the fact that BBS is a rare disease and patient numbers are limited, making recruitment for such studies difficult. Another explanation why no impact on hippocampal function was found in Bbs6 or Bbs8−/− mice might be due to local distributions of either protein: Bbs4−/− mice showed affected dendritic spine morphology of hippocampal neurons [62]. Interestingly, ciliopathy phenotypes seem not to be identical across neuronal tissues. For example, in Bbs4 mutant mice, mostly neurons in hippocampus and amygdala were affected, while other brain areas showed no changes [63]. However, in Bbs8 mutant mice, astrocytosis was described at one month of age, but seems to occur more widespread, as indicated by increased GFAP immunoreactivity in the hippocampus, corpus callosum, striatum, hypothalamus, and subventricular zone [47]. However, it seems not to affect hippocampal function at two months of age. For Bbs6−/− mice, we were not able to identify a respective report about the locality of effects.

Astonishingly to us, even though the Bbs6 and 8−/− mice displayed reduced social dominance, this seemed not to be based on a generalized increased anxiety. Even so, the animals were found to have a higher latency in the forced swim test, which became statistically significant in the Bbs8 knockout animals. Additionally, time to enter the brightly lit department in the neophobia test was decreased and time to grab food in a novel environment was also decreased. It cannot be excluded that olfactory deficits even delayed grabbing of palatable food in the NISOF test and have thus prolonged measured time span. Similarly, the onset of visual deficits might have positively influenced performance in the neophobia test, as a strong light is used as an aversive clue. If animals have reduced visual capacity, this might not be perceived as distracting, as is the case for wild type mice. Nevertheless, together with the increase in distance travelled and decreased time in corner zones in the open field paradigm, these results lead to the conclusion that mice lacking Bbs6 or Bbs8 have reduced anxiety. This contradicts data regarding Bbs4−/− mice, which showed increased levels of anxiety as measured by decreased center to total distance ratio in the open-field test and fewer light–dark transitions in the light–dark box test [13]. 

The underlying reason for this discrepancy has yet to be unraveled. However, we assume that it lies within the different functions of each of the BBS proteins. Although Bbs4 and Bbs8 are both components of the BBSome, the two proteins are not completely overlapping and might even have different alternative functions. Another explanation might be given by the age of the animals tested: the Bbs4 knockout mice were two months old, while the animals tested in our study were predominantly younger (four to eight weeks). Behavior in adolescence and during the demand of a still growing and developing brain might deviate from those of adult individuals. Although sexual maturity in mice is achieved between eight and 12 weeks, brain development still progresses up to three months (myelination, flattening of cortex). Thus, even eight-week-old mice can probably not be seen as fully matured in regard of brain function [64]. Reports on anxiety, fear, or depressive behavior in patients could not be identified to further evaluate the importance of our findings of reduced anxiety in the mice lacking Bbs6 or 8.

A behavioral test with a certain vagueness of interpreting the outcome is the marble burying test, which is thought to measure anxiety, but is also seen as a test for obsessive-compulsive behavior as found in autism spectrum disorders. For human BBS patients, evidence for autistic behavior has been described: Kerr and colleagues found autistic features in 77% of study participants [61], but studies are scarce. Bbs6−/− mice showed increased marble burying activity, while this was decreased in the Bbs8−/− mice. The increased activity would indicate an autistic trait, but the reduced activity in the Bbs8−/− mice can also be interpreted as reduced anxiety towards the novel objects. Further, it must be noted that different mouse strains show different levels of inherent digging activity (reviewed in e.g., [28]). Nevertheless, we here controlled for the respective corresponding wild types and also ascertained a lack of locomotive deficits.

In summary, while all other tests displayed a similar effect in both knockout strains with a more pronounced effect in Bbs8−/− mice, the marble burying test showed clear differences. The observed internalizing social behavior of the mouse lines reflects the symptoms of patients, while cognitive deficits are not recorded at this early age.

### 3.3. Effect of BBS Deficiency on Hypothalamic Neurons

We assumed that an altered anxiety behavior might be based on altered levels of corticosterone, thus, we weighed adrenal glands and measured the sterol in serum. However, no differences were observed. Assessing the hormone status of young male BBS patients concluded that a defect of the hypothalamic-pituitary-system might be underlying hypogonadism [58]. Previously a study using iPSC-derived hypothalamic neurons from homozygous BBS1 M390R and BBS10 C91fsX95 mutation and heterozygous BBS10 S303RfsX3 mutation carriers identified the role of the BBS proteins as a central hub for energy metabolism (comment from [65] on [66]). Here, we showed that hypothalamic neurospheres derived from Bbs6 knockout mice displayed reduced calcein stain, which points at reduced viability, but increased size, indicating altered growth behavior or metabolism. We did not assess other markers of proliferation or counted cellular numbers, thus, we cannot further elucidate the underlying mechanism of this altered neurosphere behavior. Additionally, regarding the in vitro growth properties of hypothalamic neurospheres, the hypothalamus of Bbs6 and 8−/− mice showed reduced Lsamp and Adam10 mRNA levels. Lsamp belongs to the IgLON family, including furthermore, e.g., OBCAM and Negr1, which play important roles in axonal extension, arborization of dendrites, and developmental synaptogenesis (e.g., [34,67,68]). Lsamp and neurotrimin were identified as Adam10 substrates in dorsal root ganglia [33] and shedding promoted neuronal outgrowth. Interestingly, Lsamp deficient mice showed a behavioral phenotype resembling that found in the Bbs6 and 8−/− mice: they responded with increased activity to novelty, they were less anxious, and had reduced dominance behavior [69,70,71]. The Lsamp gene makes use of two different promoters with the 1a promoter being active in limbic structures with highest expression in hippocampus and amygdala, while 1b has been found to be active in thalamic sensory nuclei, cortical sensory areas, but also in stress and arousal-regulating areas [72]. Only two hypothalamic nuclei, the paraventricular nucleus and mammillary bodies, displayed activity for both 1a and 1b promoters. It has been assumed that Lsamp is involved in emotional and social operating systems by a complex regulation of usage of the two promoters, however, our qRT-PCR does not resolve for these isoforms so that we cannot decide if one of the resulting isoforms is more affected in either mutant. Nevertheless, it is of note that, in neuropsychiatric disorders, reduced expression of human LSAMP has also been described [73], and certain SNPs within the gene have been found to be associated with major depressive disorder [74]. Reduced shedding of the GPI-anchored protein due to reduced expression of the sheddase ADAM10 might further corroborate its function. Neither LSAMP nor ADAM10 have, to the best of our knowledge, yet been described in correlation with BBS, nor with primary cilia. However, as Notch processing is based on ADAM protease activity (for example [75]), and Notch signaling is dependent on primary ciliary structure (reviewed e.g., in [76]), this might be the mechanism by which ciliopathy affects not only the hypothalamic neurospheres/hypothalamic function but, also, finally, behavior. We cannot exclude that other hypothalamic genes and proteins might have contributed to the observed behavioral phenotypes, as we only analyzed selected genes. Another limitation can be seen in the fact that we did not assess protein levels and, thus, can only speculate on functional consequences. However, regulators of ADAM10 expression have been described in the context of Alzheimer’s disease, where the protease exerts beneficial effects. For example, the synthetic retinoid acitretin was able to elevate ADAM10 mRNA levels, protein levels, and activity in mouse models and, finally, also in human patients [77,78]. If ADAM10 and Lsamp can be confirmed as major players in ciliopathy-associated behavioral phenotypes in future studies, this might be a suitable way to ameliorate behavioral deficits in BBS model mice and patients.

## 4. Materials and Methods

### 4.1. Animals

The generation of Bbs6/Mkks6 and Bbs8/TTC8 knockout mice was previously described [10,11]. For the purpose of this study, Bbs6 knockout mice were maintained on a homogenous J129 background, and Bbs8 knockout mice were crossed to BALB/canNRj mice. Homozygous Bbs6/Mkks6−/− and Bbs8/TTC8−/− knockout mice and Bbs6/Mkks+/+ and Bbs8/TTC8+/+ wild type littermate controls were selected by genotyping from heterozygous crosses. Genotyping was performed, as previously described. All animal experiments were carried out in accordance with the recommendations of the European Communities Council Directive regarding care and use of animals for experimental procedures and were approved by local authorities (Landesuntersuchungsamt Rheinland-Pfalz; approval number G 19-1-025).

### 4.2. MRI and Volumetrics

The MR imaging sessions were conducted at the Mainz Animal Imaging Center (MAIC). Animals were subjected to MRI under isoflurane anesthesia using a 9.4 T small animal imaging system with a 0.7 T/m gradient system (Biospec 94/20, Bruker Biospin GmbH, Ettlingen, Germany), as described before [79]. To investigate the size of the ventricles, the following imaging protocol consisting of two T_2_-weighted sequences was used after homogenization of the B_0_-field in the brain volume. The first sequence measured was a 2D TurboRARE sequence with high T_2_ weighting and low resolution. The parameters were (TR/TE) = (7700/100) ms, 12 averages, 30 echoes in the echo train, (0.1 × 0.1) mm^2^ resolution, 9 slices measured with a slice thickness of 0.7 mm (0.3 mm gap), and a (192 × 180) acquisition matrix. Then, a high-resolution 3D TurboRARE sequence was measured with the following parameters: (TR/TE) = (1800/32) ms, 1 average, 10 echoes in the echo train, (0.78 × 0.78 × 0.313) mm^3^ resolution, and a (258 × 120 × 48) acquisition matrix. Brain and ventricular volumes were quantified using 3DSlicer software (version 4.10.2, https://www.slicer.org, accessed between 30 June 2020 and 12 November 2020). Segments were defined using the segment editor module, followed by construction of the 3D model using the model maker tool available in the software. Finally, the volumes of the brain and ventricles were evaluated using segment statistics.

### 4.3. Behavioral Tests

Behavioral tests, where a video-based analysis was needed, were conducted using the software ANY-maze (version 6.1; Stoelting Europe, Dublin, Ireland) and a digital camera (Imaging Source, Bremen, Germany). All tests were started after a 15 min habituation time for the animals after being brought to the test room. Animals have been single-caged before assessing sexual behavior so as to avoid influence of dominant/submissive state.

- Pole test

Mice were placed on top of a 55 cm high metal pole with the head upwards. Time was stopped when the mouse reached the floor (a bedding material filled tub) with all four paws. Each mouse was tested twice and the mean of values was calculated. A maximum time span of 120 s was set if the mouse did not reach the floor earlier or if it dropped.

- Olfaction test

The test for olfaction was conducted, as described previously [24]. In brief, the mice were habituated to palatable food (yellow fruit loop, Kellogg’s, Kellogg Europe Trading Limited, Dublin, Ireland) and subsequently had to search for buried entities of the food. The time until the mouse grabbed and gnawed on the food were measured.

- Huddling test

Huddling of neonatal mice allows more ecological thermoregulation; however, huddling behavior continues into adulthood in rodents due to social interaction (filial huddling, [80]). The percentage of mice huddling together in one quadrant of the cage from all animals asleep was assessed at 4 p.m.

- Social interaction

Mice were exposed to an empty cylinder (18 cm × 7.5 cm) within an open field arena (60 cm × 60 cm). Subsequently, the cylinder was presented with non-familiar mice of same sex and age—C57Bl/6J OlaHsd mice (Envigo, Horst, Netherlands; two different animals used per to be tested mouse). On the following day, each mouse was exposed to the cylinder containing each of its cage mates. SI scores were calculated by dividing time in zone (8 cm around the cylinder) with the conspecific divided by time spent in zone with the empty cylinder.

- Tube test

In brief, a plastic tube with a diameter of not allowing passing of two mice in opposite direction was used to measure dominance of the mice. Mice were tested with cage-mates, as described previously [81].

- Urine Scent Marking test

Urine scent marking as an assessment of sexual interest has been described previously [24]. In brief, female wild type urine from pro-estrous state was spotted on white paper within an open field arena and the male test mouse was inserted. After 10 min the urine that the male has voided was stained by ninhydrin solution and signal intensity in different areas was measured after conversion into black and white image. Deviating from the former report, we here measured urine spots in three zones instead of four quadrants.

- Pasta gnawing test

The mouse was habituated to spaghetti 2 h before the test by inserting a piece and removing other kind of food. Then, the mouse was inserted with its home cage without lid, water, and paper towel into a soundproof box. A condenser microphone was installed above the cage and a 3 cm piece of spaghetti provided. The sound was recorded for 5 min using the software Audacity. Bites per bite events (event: minimally 3 bites) and bite speed were analyzed by using Avisoft software (Avisoft Bioacoustics e.K., Glienicke/Nordbahn, Germany).

- Ultra sonic vocalization assessment

Ultra-sonic vocalization was measured, as described in [82]. In addition to low frequency calls (<30 kHz), we also investigated high frequency calls (>30 kHz). On the first day, response to neutral, unused bedding material was recorded (habituation). On the second test day, bedding material from a cage of the opposite sex was inserted and, on the third day, bedding from same-sex mice was inserted. All recordings were conducted for 5 min. The distinction of high and low frequency calls was done by using Avisoft software, and call numbers were assessed.

- Nest building

The nest building assessment has been described previously (e.g., [83]). In brief, mice were provided a special nest building material after an overnight deprivation of such and, the morning thereafter, nests were scored (the higher the number, the better the nest quality). Additionally, the unused material was weighed.

- Radial Arm Water Maze

The radial arm water maze was conducted as described in [84].

- T-Maze

The T-Maze test was conducted as described in [84].

- Forced Swim test

A 3l beaker was filled with 25 °C warm water. The mouse was introduced to the beaker and the time measured until the mouse stopped swimming for at least 2 s (ANY-maze software). The animal was subsequently warmed under a red light before being brought back to the home cage.

- Neophobia test

A plastic box (10 cm × 10 cm) with a lateral small opening was inserted in the open field arena, which was brightly lighted by a strong light source (halogen floodlight, RITOS type 6095115 AIP44, 150 W, Ritter Leuchten GmbH, Mömbris, Germany), as described previously [84]. The opening of the box was closed by a plastic lid, and the mouse habituated to the box for 15 s. Then, the lid was lifted and the time was measured until the mouse entered the arena (with all four paws outside the box).

- Novelty induced repression of feeding test

This test was performed, as previously described [84]. In brief, mice were exposed to palatable food (see olfaction test) to habituate to it. Subsequently, on the test day, the mouse was inserted in the opposite corner of a palatable food piece in an open field arena (60 cm × 60 cm), and time was measured until the mouse reached for the food in the unfamiliar environment and started to feed.

- Marble burying test

Twenty black marbles were lane-wise (5 per lane, 2.5 cm distance between each marble) inserted on the surface of fresh bedding material (400 g/cage). After 5, 10, and 15 min, the number of buried marbles was assessed. A marble was designated as buried if at least ¾ of it was covered by bedding material.

- Open field-assessed parameters

An open field arena that measured 60 × 60 cm was used. An inner center of 16 cm diameter was defined, as well as the corners (8 × 8 cm). Each mouse was investigated for 15 min. Distance travelled and time spent in the corner zones was recorded and analyzed by ANY-maze. The data shown correspond to the first 5 min.

### 4.4. Dissection

Animals were anesthetized with isoflurane (Piramal, Mumbai, India) and decapitated. Truncal blood was collected and serum prepared by two centrifugation steps (10 min at 10 °C, 680× *g* and 15 min at 10 °C, 680× *g*). The brain was removed and the hypothalamus collected in RNA later (Qiagen, Hilden, Germany). Adrenal glands were collected in pre-weighed tubes and weight measured of the pair of organs. Samples were stored at −80 °C until further use.

### 4.5. Corticosterone Measurement in Serum

Serum samples were collected, and corticosterone quantification was performed, as described before [82]. In brief, serum was mixed with a dexamethasone (Cayman Chemicals, Ann Arbor, MI, USA) standard and a fixed amount of corticosterone (Sigma Aldrich, St. Louis, MO, USA) to monitor and facilitate measurement of the endogenous levels. Samples and standards were extracted two times and subjected to reversed-phase HPLC analysis. The HPLC (automatic liquid autosampler and isocratic pump: Series 1100; thermostatted column compartment and variable wavelength detector: Series 1200, all Agilent Technologies, Waldbronn, Germany) was equipped with an ODS Hypersil C18 column (5 µm, 150 mm × 4 mm; MZ-Analysentechnik, Mainz, Germany) and temperature set at 24 °C. Detection was carried out at 245 nm with a consistent flow rate (1.2 mL/min). Chromatograms were obtained and analyzed by ChemStation for LC software (Rev. A.10.02, Agilent Technologies, Waldbronn, Germany).

### 4.6. RNA Preparation and qPCR

RNA was extracted from the hypothalamus (RNeasy^®^ Lipid Tissue, Qiagen, Hilden, Germany). Quantitative polymerase chain reaction (RT-qPCR) was performed using exon–exon boundary-spanning primer sequences (see below) and the SYBR Green methodology on a Step One Plus sequence amplification system (Applied Biosystems, Foster City, CA, USA). The relative mRNA expression of the tested gene normalized to Gapdh expression was calculated using the ΔΔCt method.

The primers were as follows: Negr1 forward/reverse: ATGTGACGCAGGAGCACTT/CCATACTGGGCTGTACTTGGA [85]; Lsamp forward/reverse ATCACCAGGGAACAGTCAGG/TCCCGGTACCACTCAAAGTC [67]; Adam10 (QT00106351, Qiagen, Hilden, Germany).

### 4.7. Neurosphere Culture

Briefly, the freshly isolated hypothalamus was cut into small pieces, and cells were dissociated with TrypLE Select enzyme (Gibco) for 30 min at 37 °C. The cells were filtered through a 40 µM filter (Greiner Bio-One GmbH, Frickenhausen, Germany) and then centrifuged at 300× *g* for 10 min RT without brake. Cells were suspended in medium consisting of Neurobasal-A supplemented with 0.24% GlutaMAX, 4% B27 without vitamin, 20 ng/mL epidermal growth factor (EGF), and 10 ng/mL basic fibroblast growth factor (bFGF) (all Life Technologies, Carlsbad, CA, USA). 200,000 cells per well in 600 µL medium were seeded in a 24-well plate with low adhesion. The cells were cultivated for 7 days for further analysis.

### 4.8. Calcein Staining and Neurosphere Size Determination

For the staining, 0.2 μM Calcein AM (Cayman Chemicals, Ann Arbor, MI, USA) was added to the cells in the medium. After an incubation for 15 min at 37 °C, the staining solution was removed, and the cells were washed twice with PBS. The neurospheres were visualized under the microscope (ZOE Fluorescent Cell Imager, Bio-Rad Laboratories GmbH, Feldkirchen, Germany). Fluorescence intensity was measured in lysates (Passive lysis buffer, Promega, Walldorf, Germany) of the neurospheres at 485 ex/520 em nm. For size determination, the diameter of six neurospheres per animal (measured horizontal) was determined using Fiji/ImageJ software (NIH, Bethesda, Rockville, MD, USA).

### 4.9. Statistical Analysis

All statistical analyses were performed using GraphPad Prism 6 or 8 for Windows (GraphPad Software, Suite, CA, USA). Data are graphically represented as mean + standard error of the mean (SEM) (Figure 1) or scatter plots showing individual values. Statistical analysis was performed with an unpaired two-tailed Student’s *t*-test (* *p* < 0.05; ** *p* < 0.01; *** *p* < 0.001), if not stated otherwise.

## Data Availability

All data are provided within the text or within Supplementary Materials.

## Supplementary Materials

The following supporting information can be downloaded at: https://www.mdpi.com/article/10.3390/ijms232314506/s1.
