# Supplementary material for: Behavioral Phenotyping of Bbs6 and Bbs8 Knockout Mice Reveals Major Alterations in Communication and Anxiety
Source: Int J Mol Sci. 2022 Nov 22;23(23):14506. doi: 10.3390/ijms232314506 (PMC9741393; doi:10.3390/ijms232314506)
Supplement: Supplementary file 1 [file ijms-23-14506-s001.zip › ijms-2004672-supplementary.pdf]

Table S1. Composition of groups of investigated mice (F2 generation).
Male Bbs8 +/- mice were crossed with BALB/canNRj female mice. Offspring was genotyped for albinism caused by tyr gene mutation and for knockout of the Bbs8 gene.

Bbs8-genotype	Albino-genotype	tyr^G/tyr^G				tyr^C/tyr^C		tyr^G/tyr^C			
(+/+)	#animals	6				5		11			
	sex	♂		♀		♂	♀	♂		♀	
		3		3		3	2	5		6	
	fur color	brown	grey	brown	grey	white		brown	grey	brown	grey
			3	1	2	5		1	4	4	2
(-/-)	#animals	6				5		11			
	sex	♂		♀		♂	♀	♂		♀	
		4		2		2	3	5		6	
	fur color	brown	grey	brown	grey	white		brown	grey	brown	grey
		2	2	0	2	5		1	4	2	4

Table S2. Time course of behavioral tests
Animals were housed as groups of 4 up to end of week 2, then they were single-caged.

	Week 1	Week 2	Week 3	Week 4
a.m.	Tube test<br>SI test	Marble burying test<br>T Maze test	USV recording<br>Urine scent marking test<br>Olfaction test	Olfaction test<br>Pasta gnawing test<br>Nesting test
p.m.	Huddling test<br>Neophobia test<br>RAWM	Open field test<br>NISOF test<br>Forced swim test	Pole test<br>Nesting test	Pasta gnawing test

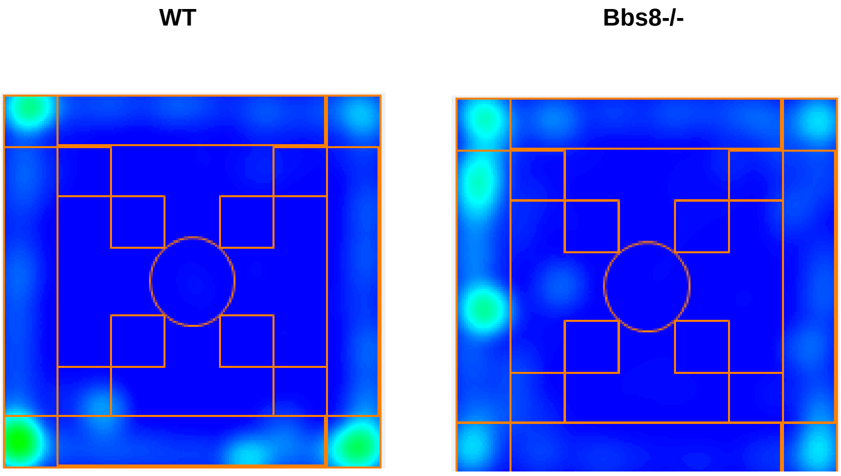

Figure S1. Examples of heat maps for movement profile visualization. A heat map reporting on the localization of an exemplary wild type and a Bbs knockout mouse is shown. The wild type animal stayed for longer periods in the corners, while the Bbs8 animal showed a more spread pattern of localization.
